# Supplementary figures and images for: Identification of novel regulators of STAT3 activity
Source: PLoS One. 2020 Mar 31;15(3):e0230819. doi: 10.1371/journal.pone.0230819 (PMC7108870; doi:10.1371/journal.pone.0230819)

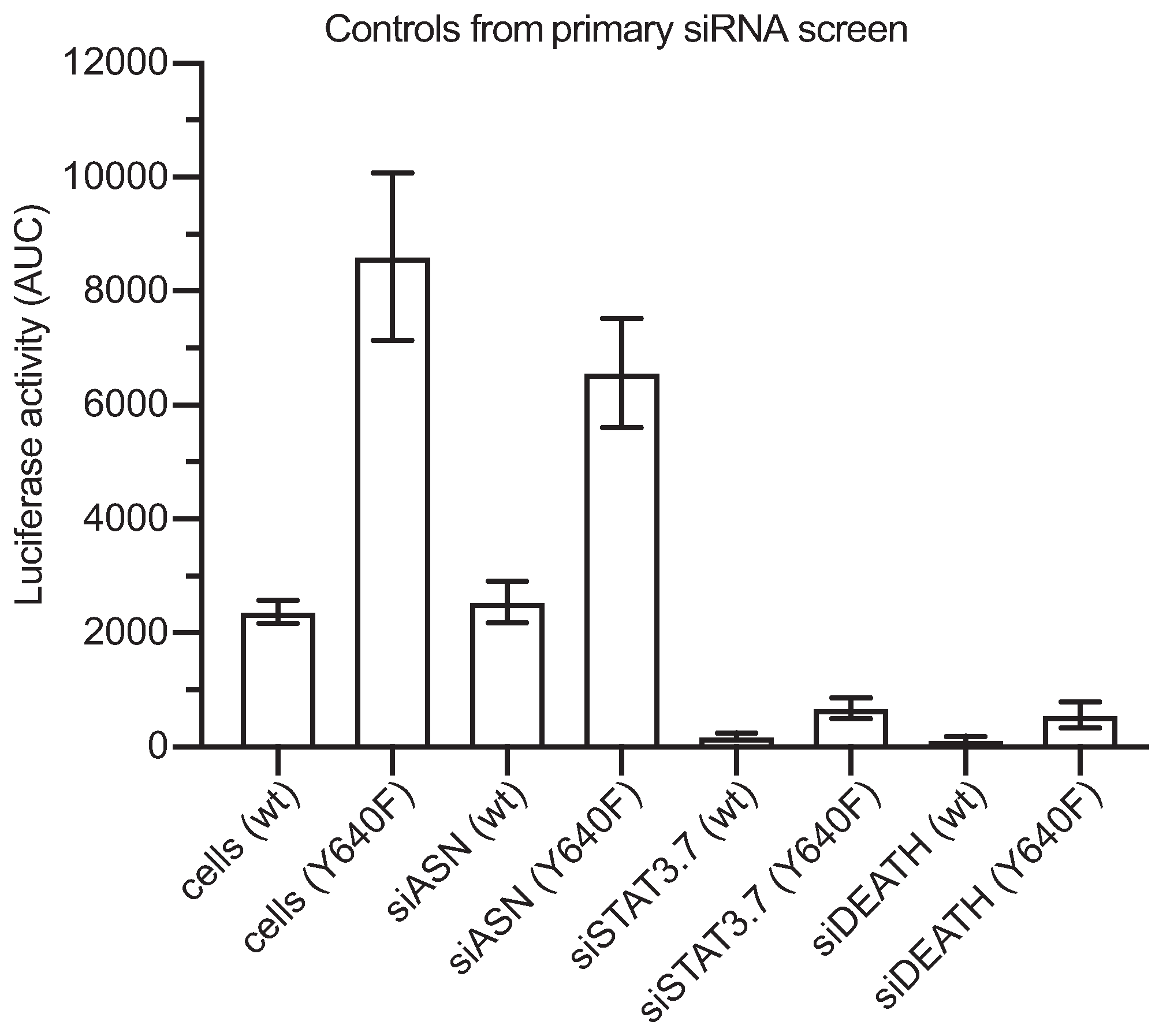

Supplement: S1 Fig — STAT3(Y640F) and IL6 induced STAT3(wt) expressing cells had 2.6-fold change in mean values of non-targeting siRNA (siASN) and 3.6-fold change in mean values of only transfection reagent (cells) treated cells. Bars represent mean and standard deviation of controls on all assay plates in one screen (total 24 technical replicates per each control). Cells and siSTAT3.7 were used to normalize the primary screen. (TIF) [file pone.0230819.s001.tif]

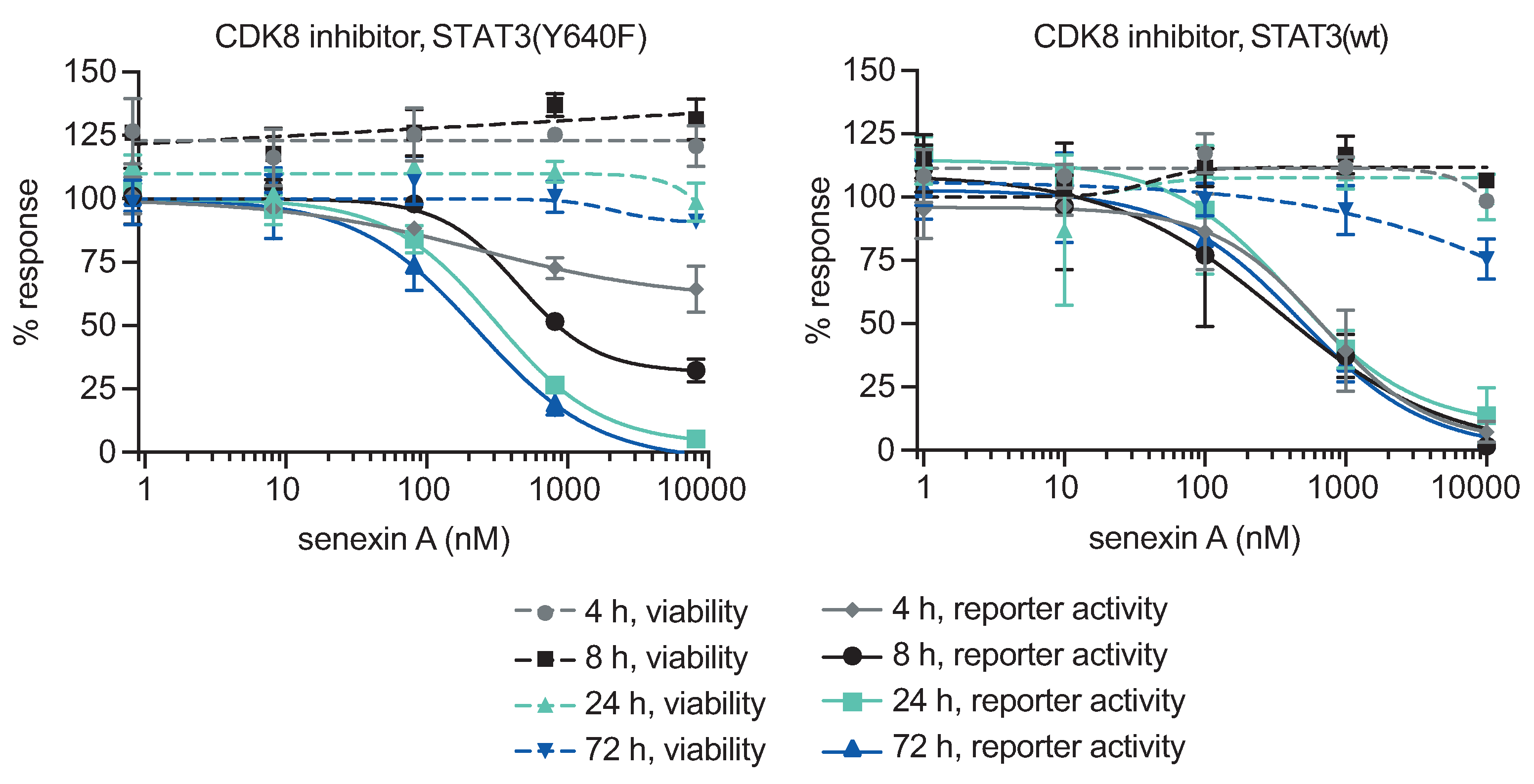

Supplement: S2 Fig — CDK8 inhibitor senexin A inhibits both STAT3(Y640F) and STAT3(wt) dose and time dependently. Dots are mean and error bars represent data from two independent experiments with two technical replicates. (TIF) [file pone.0230819.s002.tif]

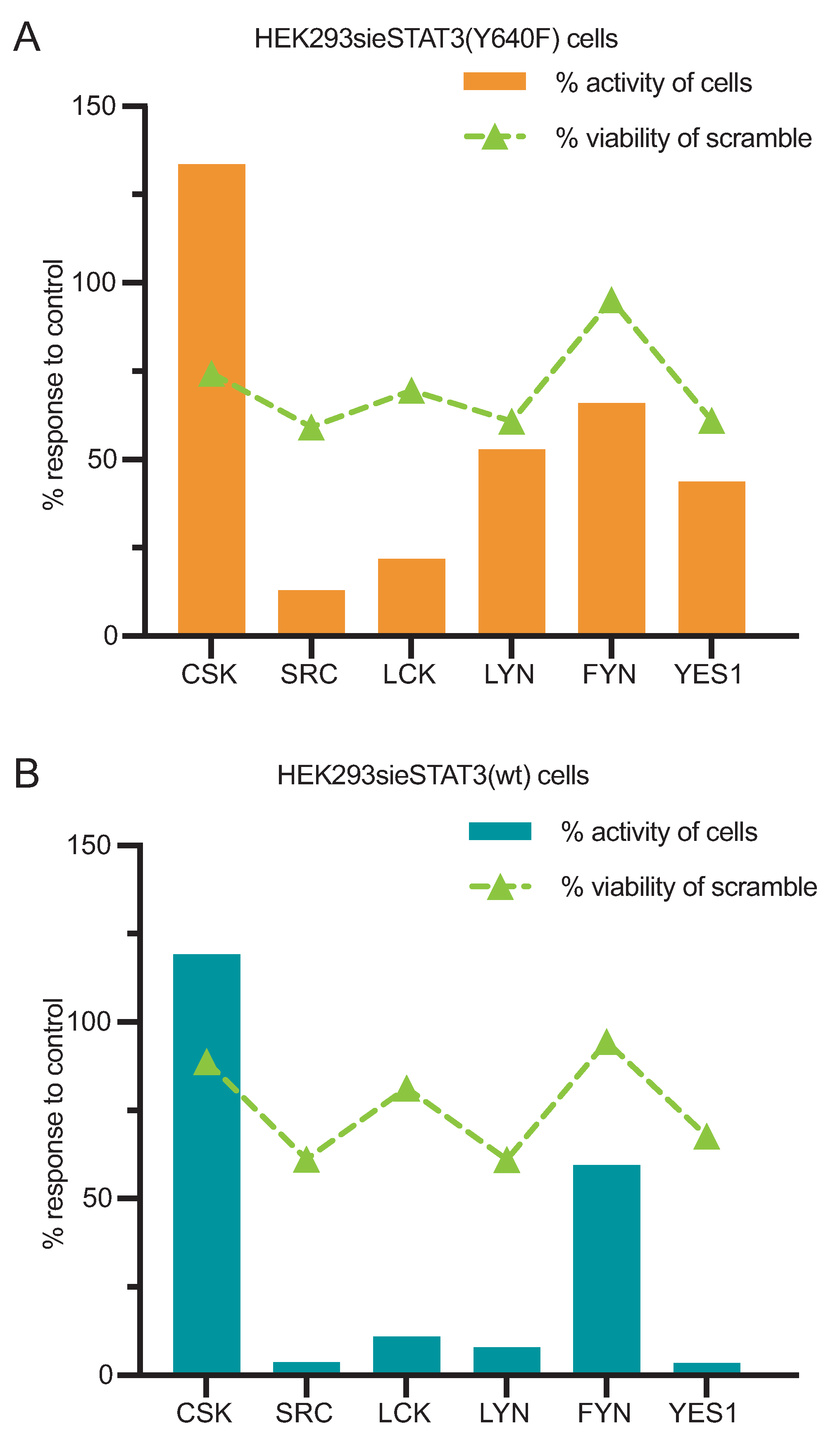

Supplement: S3 Fig — Effect of CSK and Src family kinases (SFKs) knockdowns on STAT3 reporter activity and viability of HEK293sieSTAT3(Y640F) (A) and IL6 induced HEK293sieSTAT3(wt) (B). (TIF) [file pone.0230819.s003.tif]

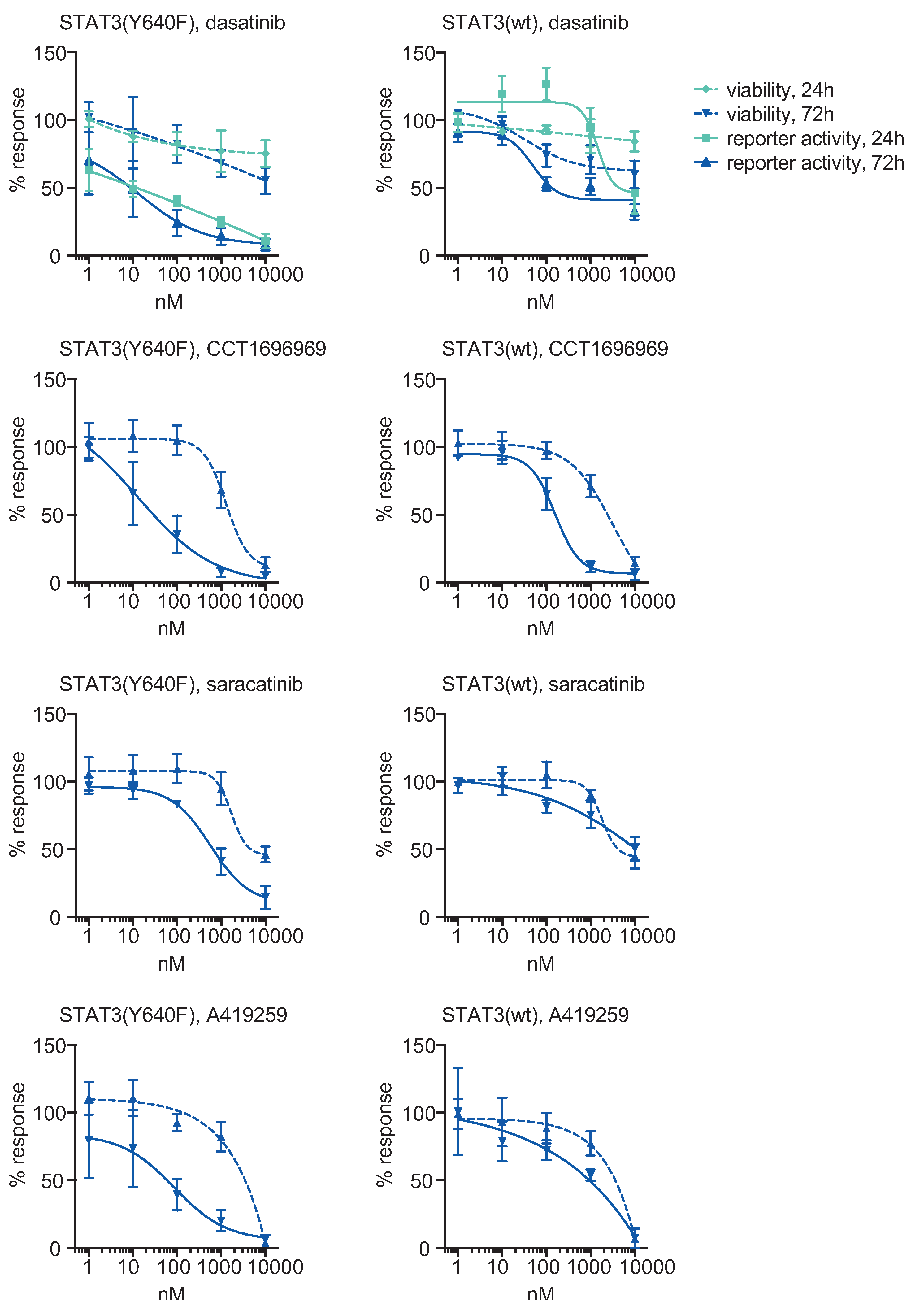

Supplement: S4 Fig — Src inhibitors (saracatinib, CCT196969, dasatinib and A419259) results in stronger reporter activity reduction after 72h in STAT3(Y640F) that in wild type STAT3 expressing. Dots are mean and error bars represent data from at least two independent experiments with two technical replicates. (TIF) [file pone.0230819.s004.tif]

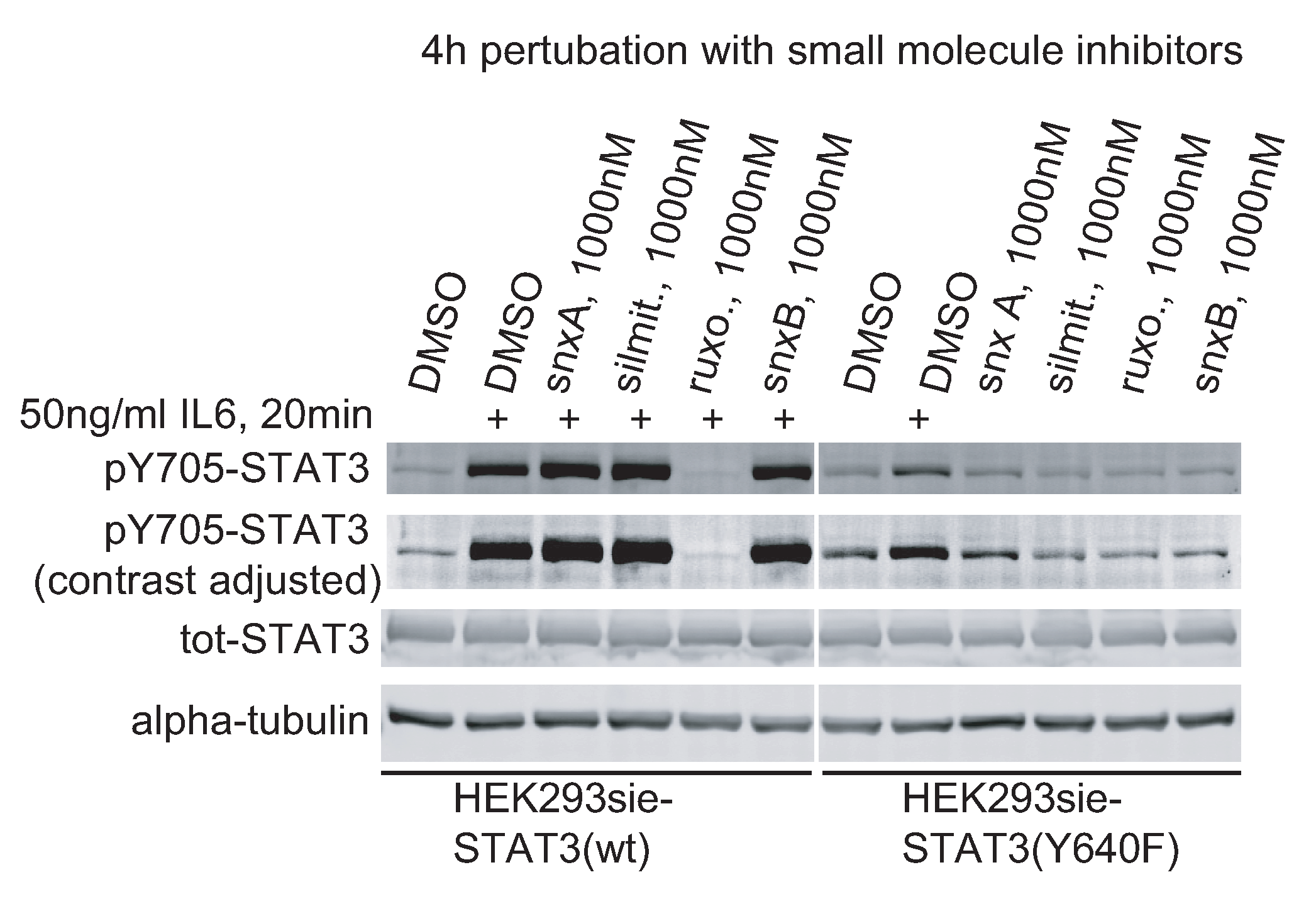

Supplement: S5 Fig — Four-hour perturbation with JAK1/2 inhibitor ruxolitinib decreases Y705-phosphorylation of STAT3 in IL6 induced STAT3(wt) expressing cells. (TIF) [file pone.0230819.s005.tif]
