# Supplementary material for: Identification of novel regulators of STAT3 activity
Source: PLoS One. 2020 Mar 31;15(3):e0230819. doi: 10.1371/journal.pone.0230819 (PMC7108870; doi:10.1371/journal.pone.0230819)
Supplement: S1 Raw images — Whole membranes of all shown and analyzed Western blot membranes with lane labelling. Lanes with “X” and/or “italics” are not included in the paper. Blots are supporting Fig 5 and S4 Fig. (PDF) [file pone.0230819.s009.pdf]

Intensity 7.5
Treatments in *italic*
not used in manuscript.
72h drug incubations

Figure panel 5 analysis

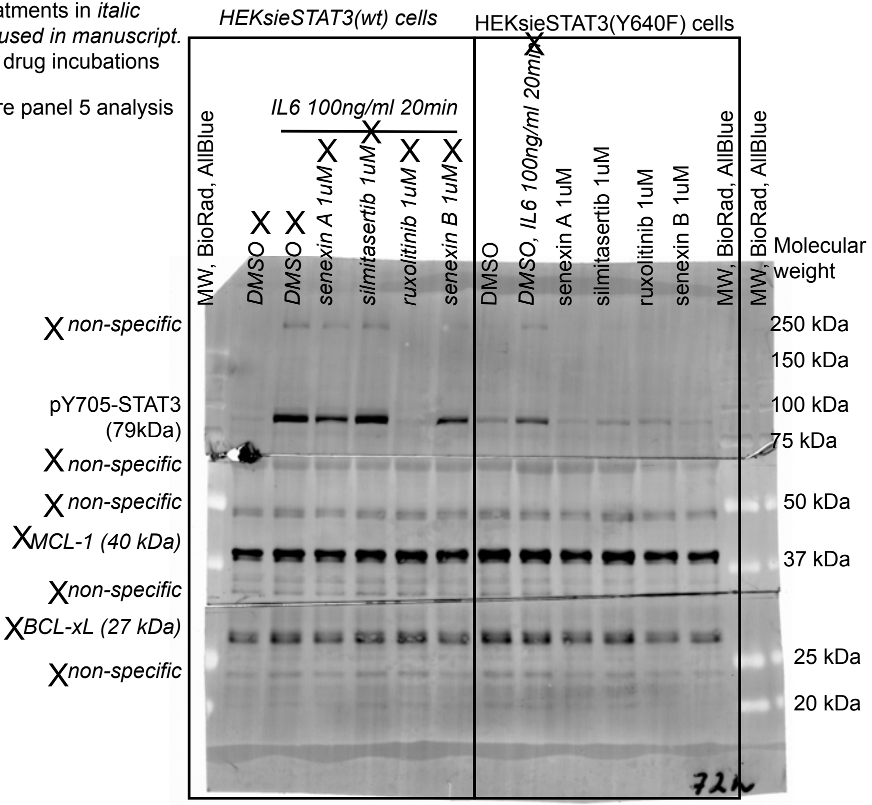

-> Loading order from left to right.

Imaging intensity 5
Treatments in *italic*
not used in manuscript.
72h drug incubations
Figure panel 5 analysis

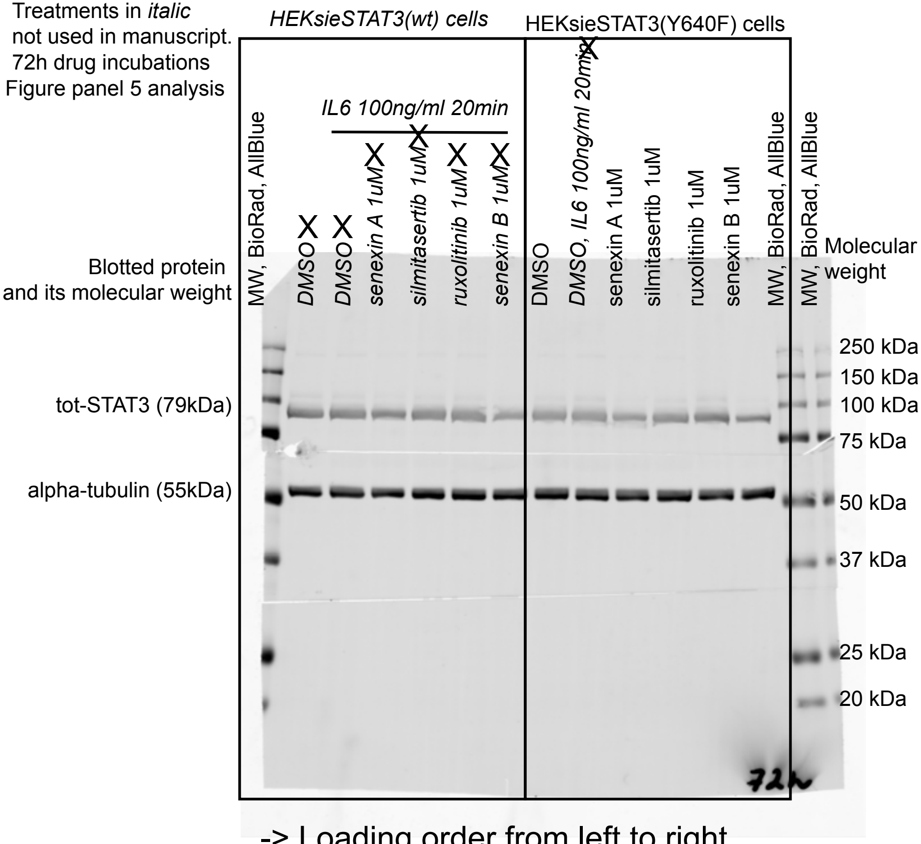

-> Loading order from left to right.

Figure panel 5 original full membrane. Imaging intensity 5.
Not used for analysis.

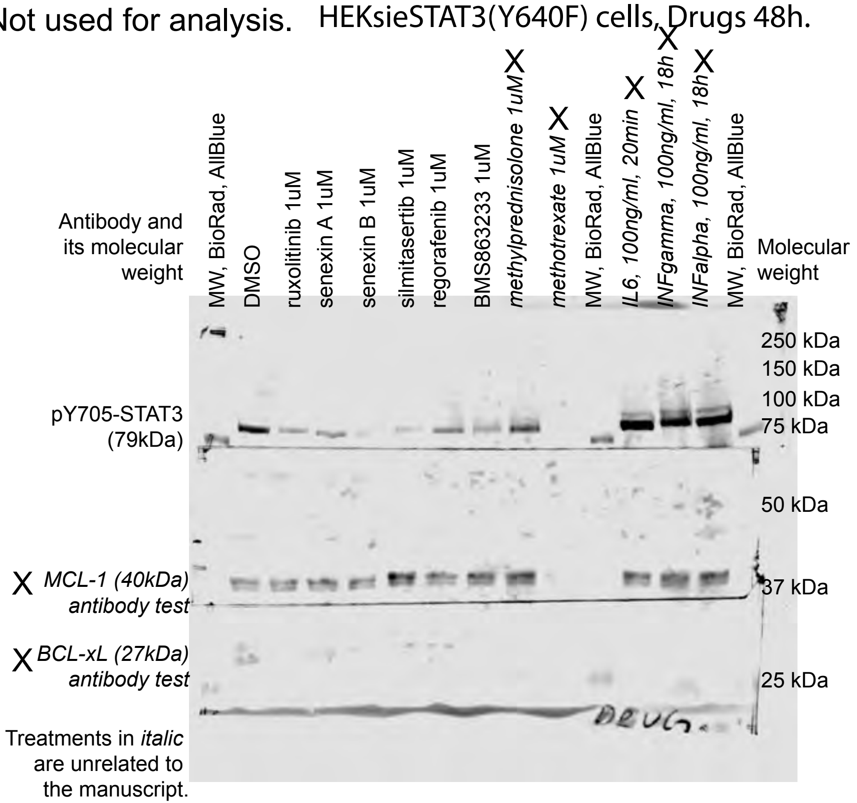

-> Loading order from left to right.

Rescan of top part of the membrane with channel 800 for pY705-STAT3.
For scanning intensity increased from 5 to 7.5.
This image was used for analysis of figure 5.

HEKsieSTAT3(Y640F) cells, Drugs 48h.

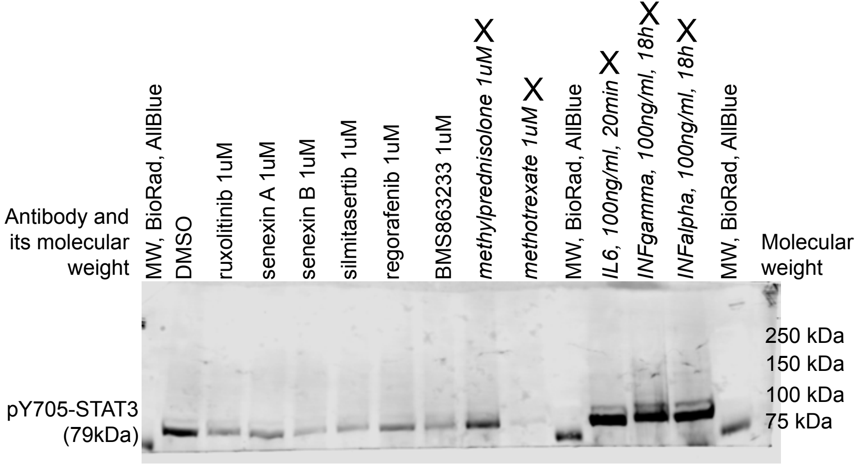

-> Loading order from left to right.

Treatments in *italic* are unrelated to the manuscript.

Figure panel 5 analysis
HEKsieSTAT3(Y640F) cells, Drugs 48h.

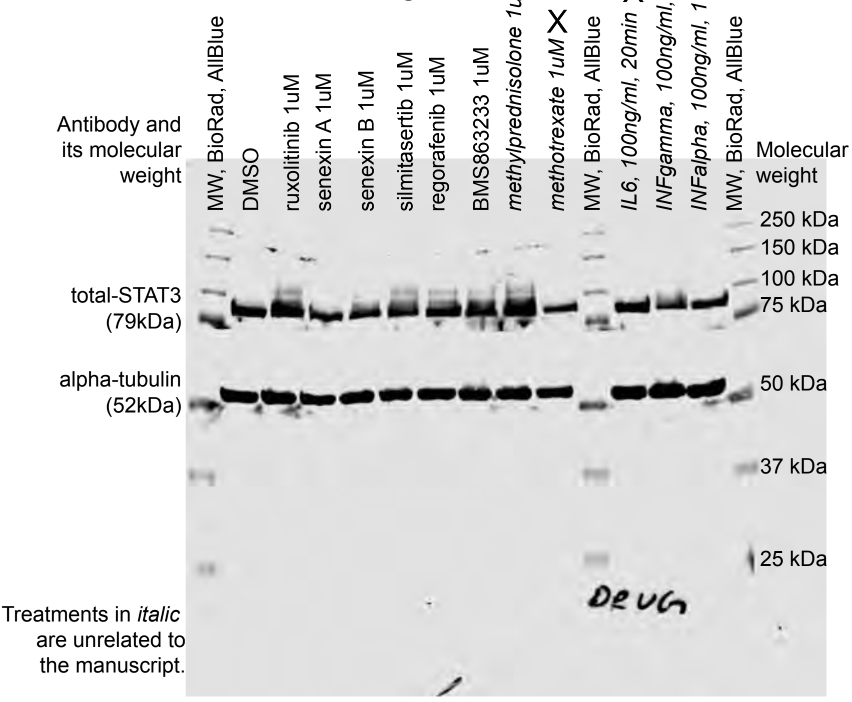

-> Loading order from left to right.

Figure panel 5 analysis. HEKsieSTAT3(Y640F) cells, Drugs 72h.

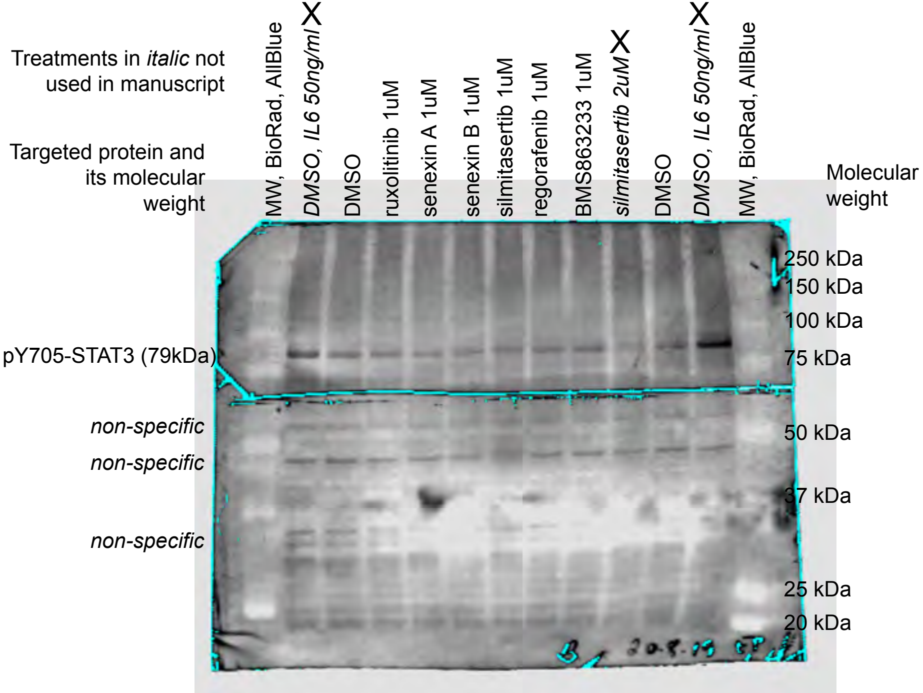

-> Loading order from left to right.

Figure panel 5 analysis. HEKsieSTAT3(Y640F) cells, Drugs 72h.

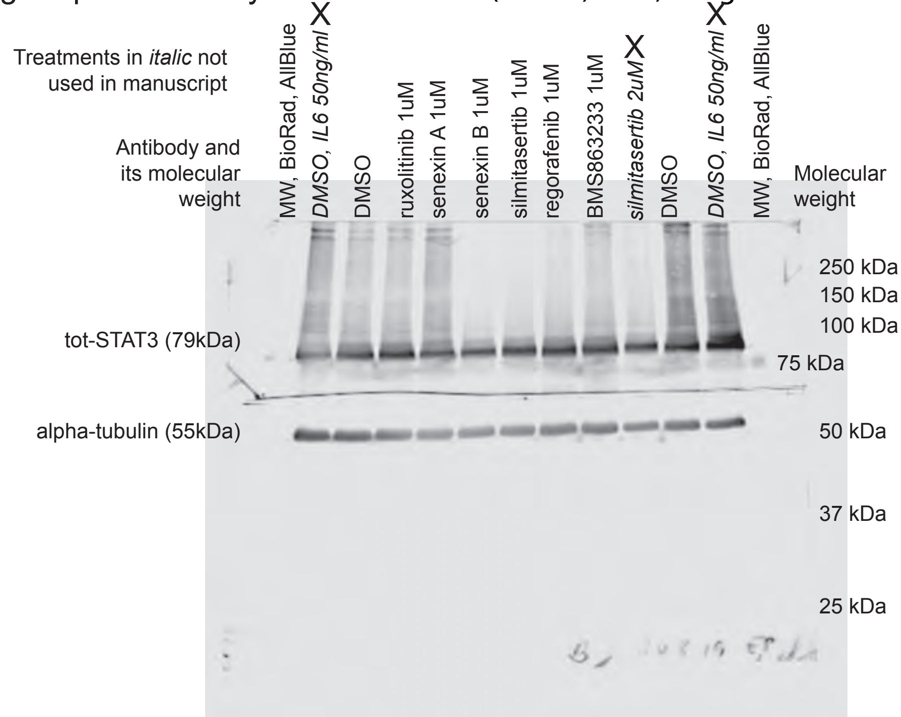

-> Loading order from left to right.

Figure panel 5 analysis and blot figure HEKsieSTAT3(Y640F) cells, Drugs 48h

Only top membrane imaged for pY705-STAT3 at intensity 7.5

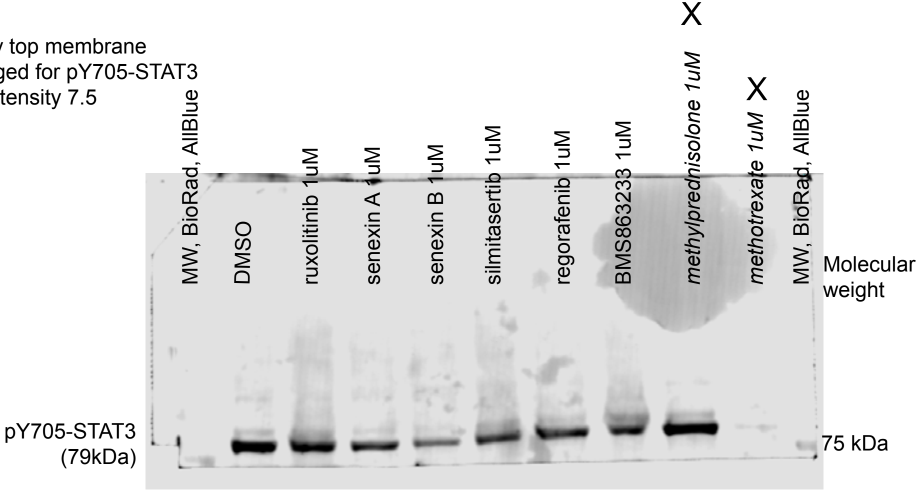

-> Loading order from left to right.

Figure panel 5 analysis and blot figure
HEKsieSTAT3(Y640F) cells, Drugs 48h
Intensity 5

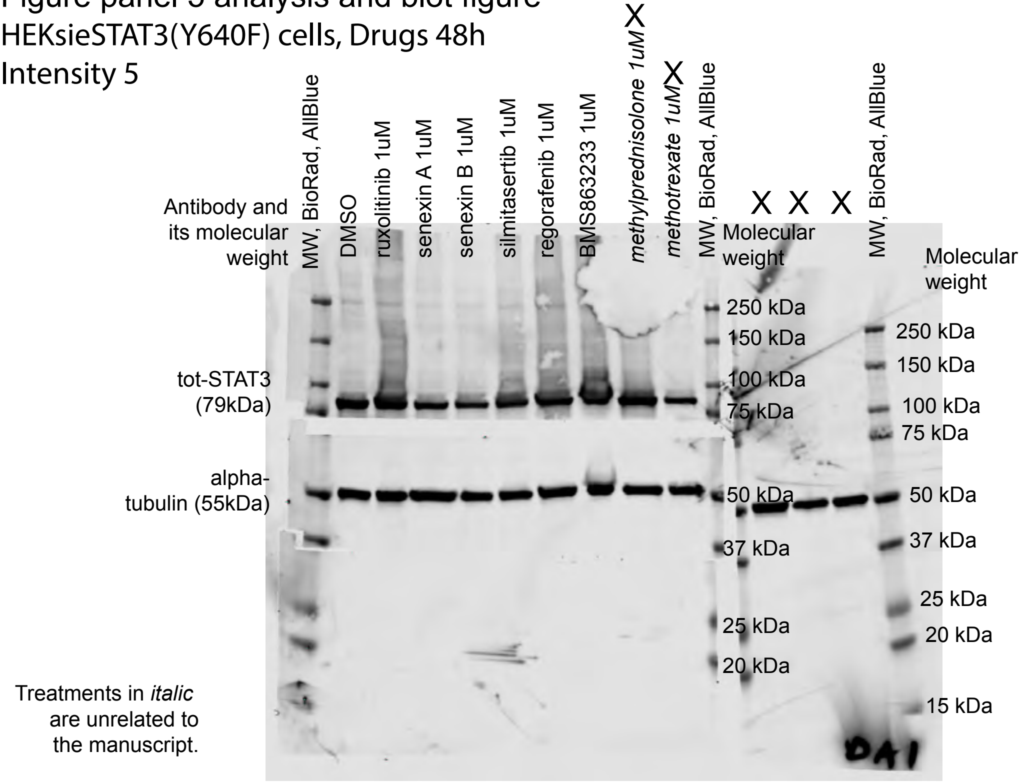

-> Loading order from left to right.

Raw image for supplementary Figure S4

Imaging intensity 7.5

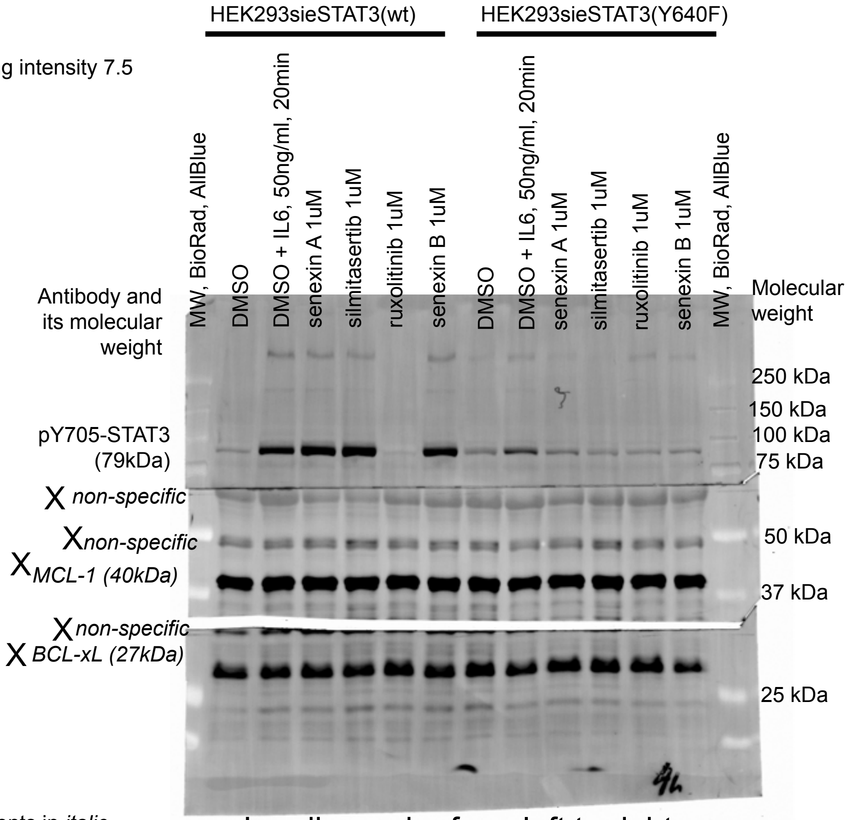

-> Loading order from left to right.

Treatments in *italic* are unrelated to the manuscript.

Raw image for supplementary Figure S4

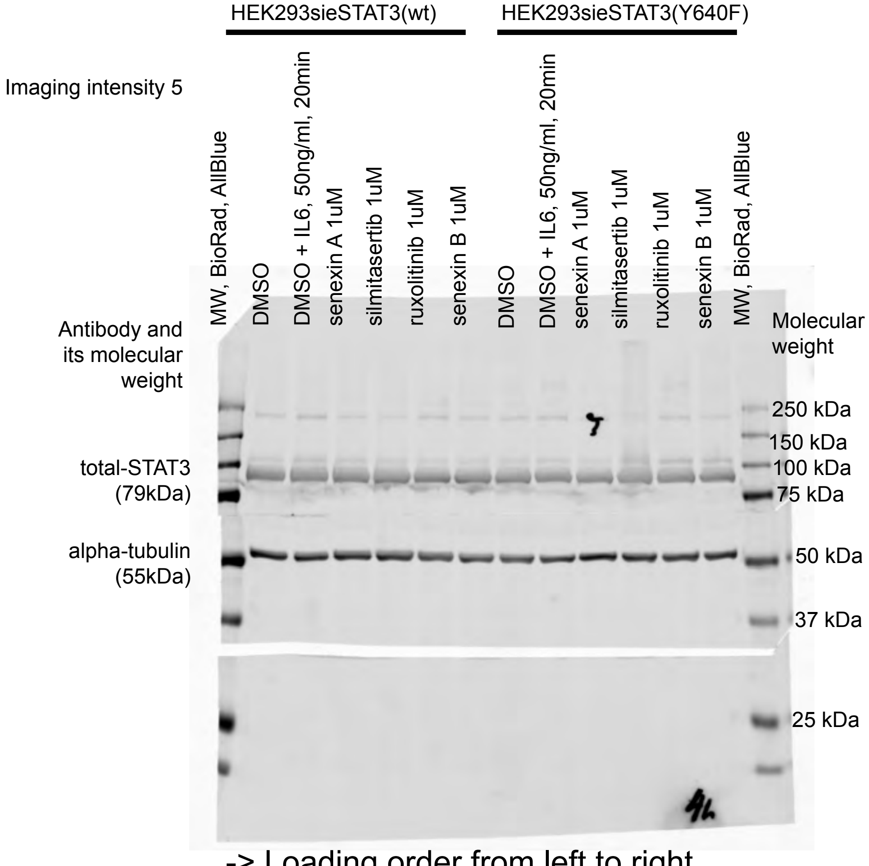

-> Loading order from left to right.

Figure 5 Analysis
HEK293sieSTAT3(Y640F) cells
Imaging intensity for pS727-STAT3 7.5
and total-STAT3 intensity 5.
Loading order left to right ->

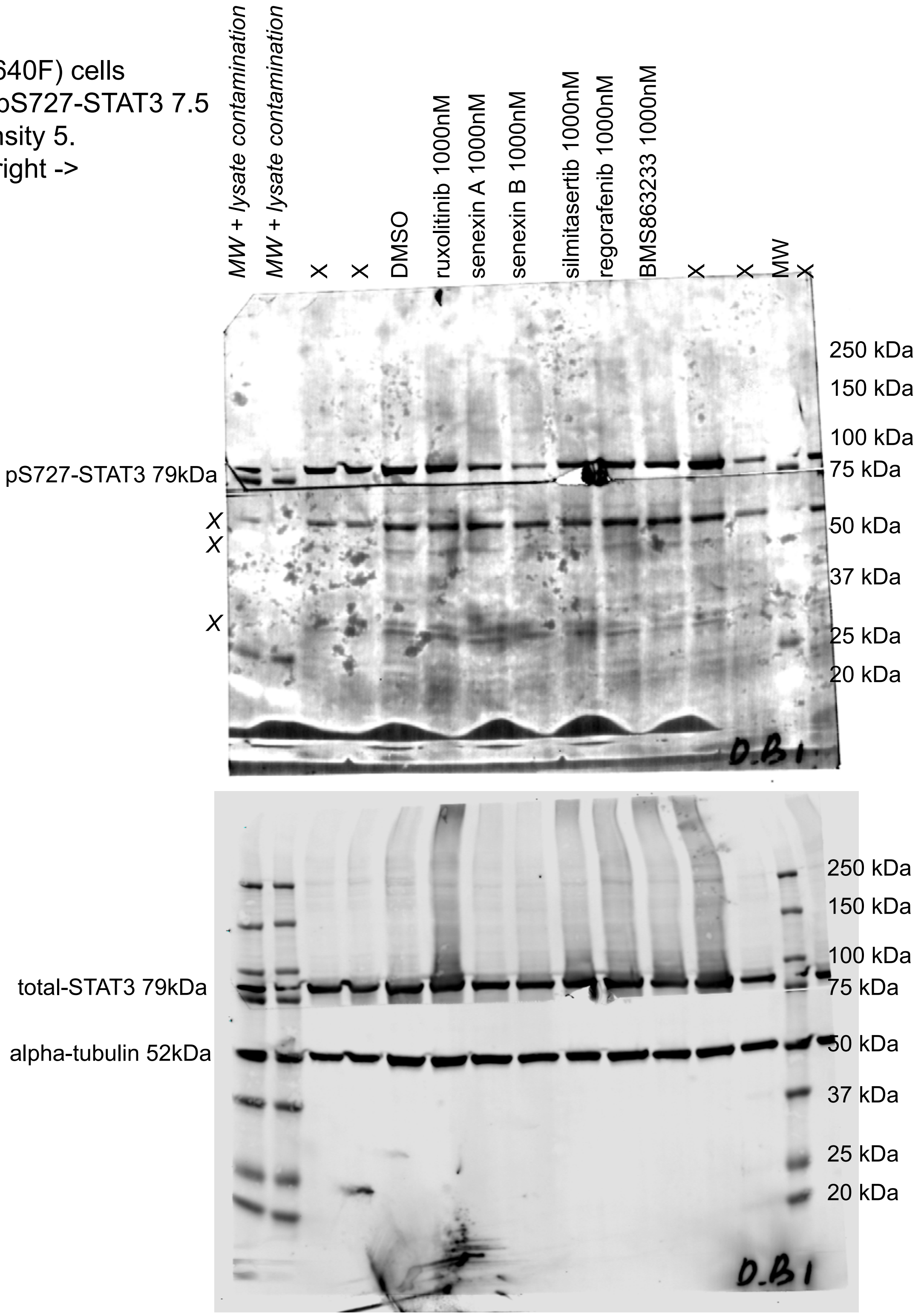

Raw images: Figure 5
Intensity 5, channel 800

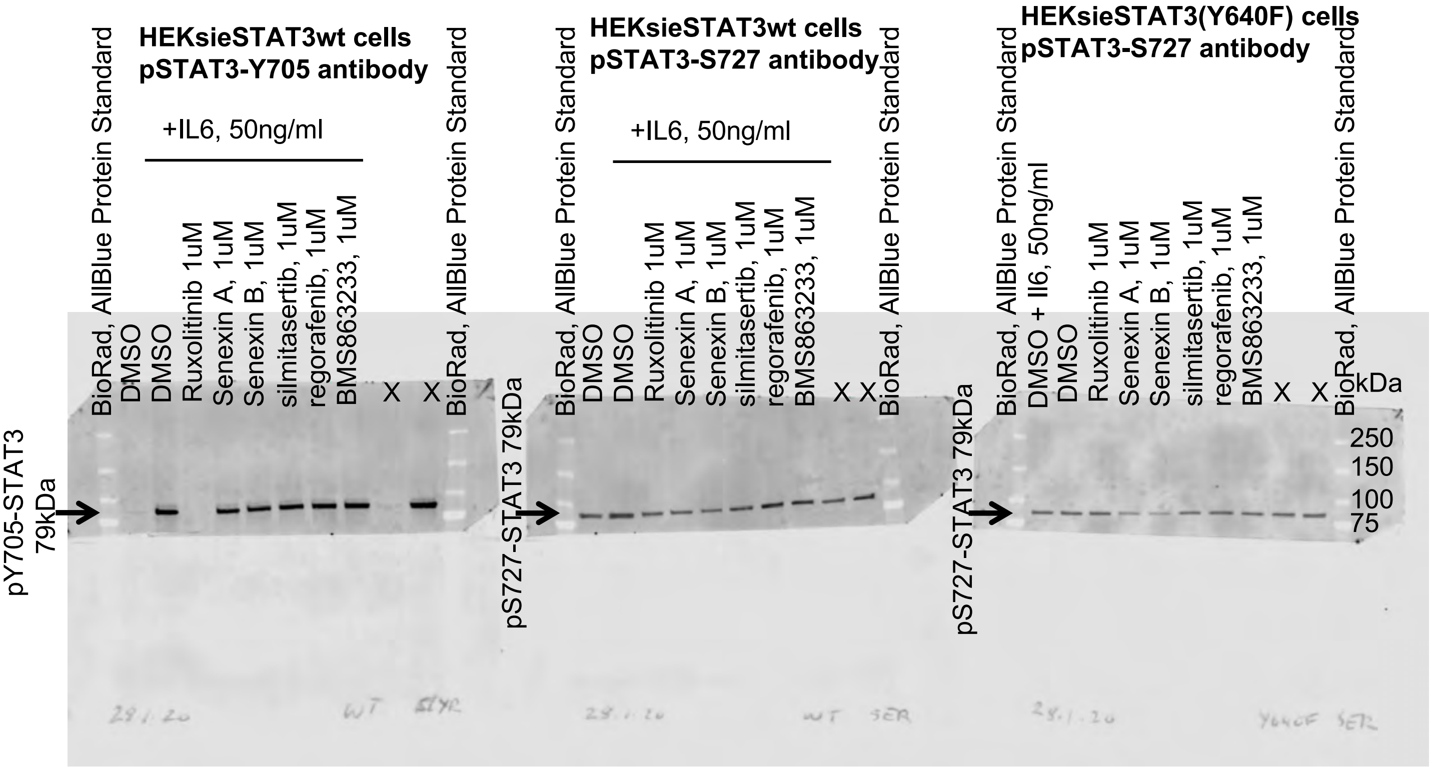

Intensity 5, channel 700

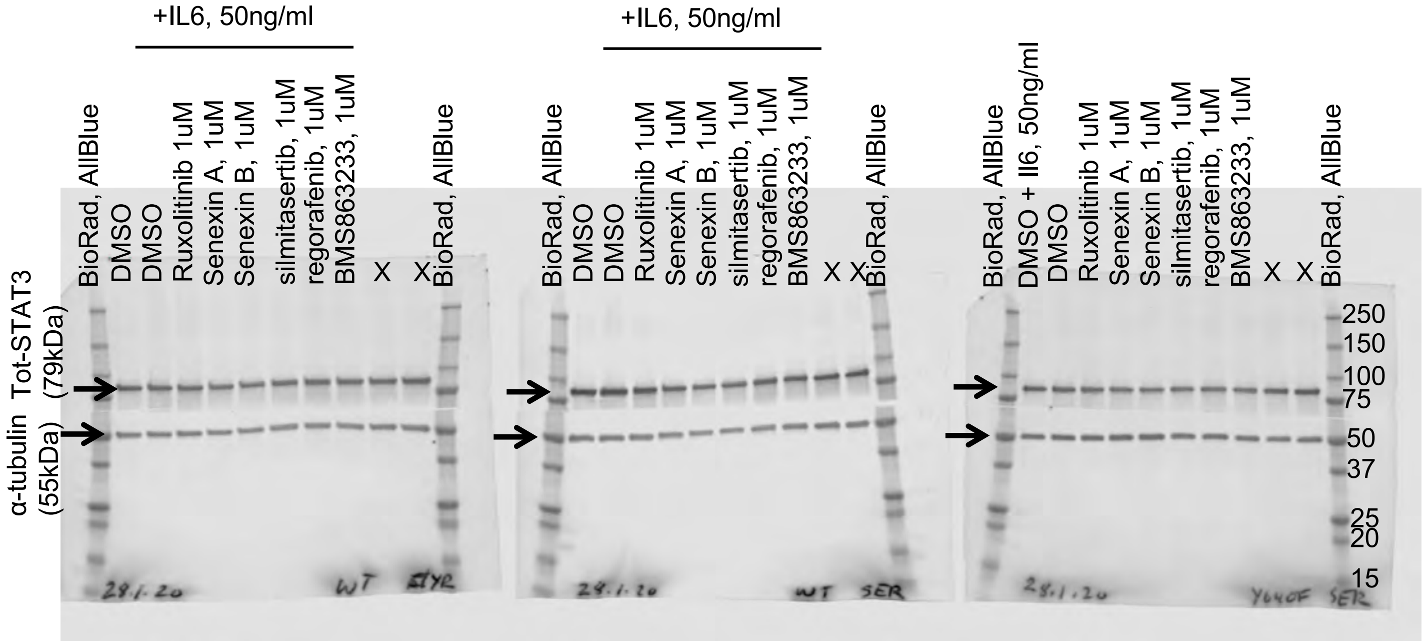

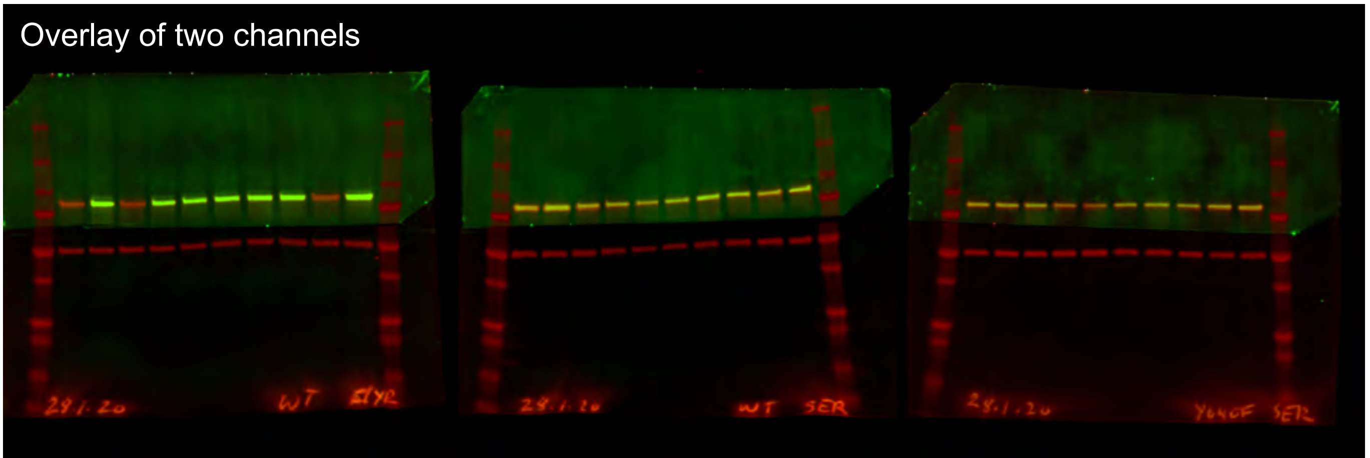

Raw images: Figure 5, analysis

Intensity 6, channel 800

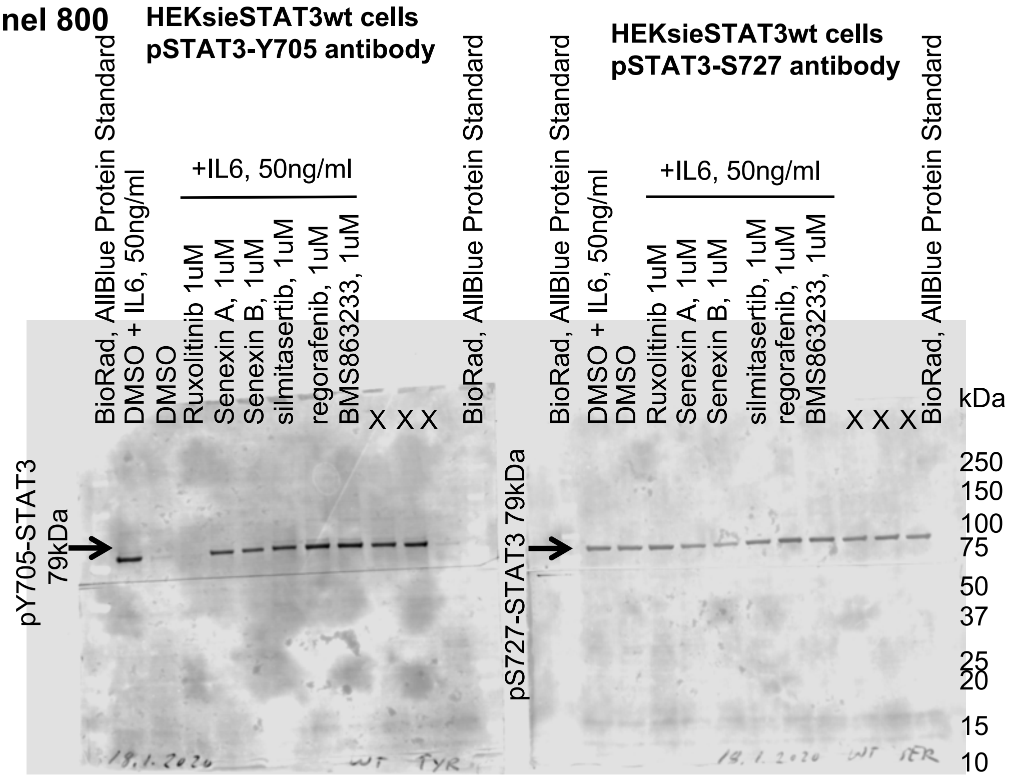

Intensity 5, channel 700

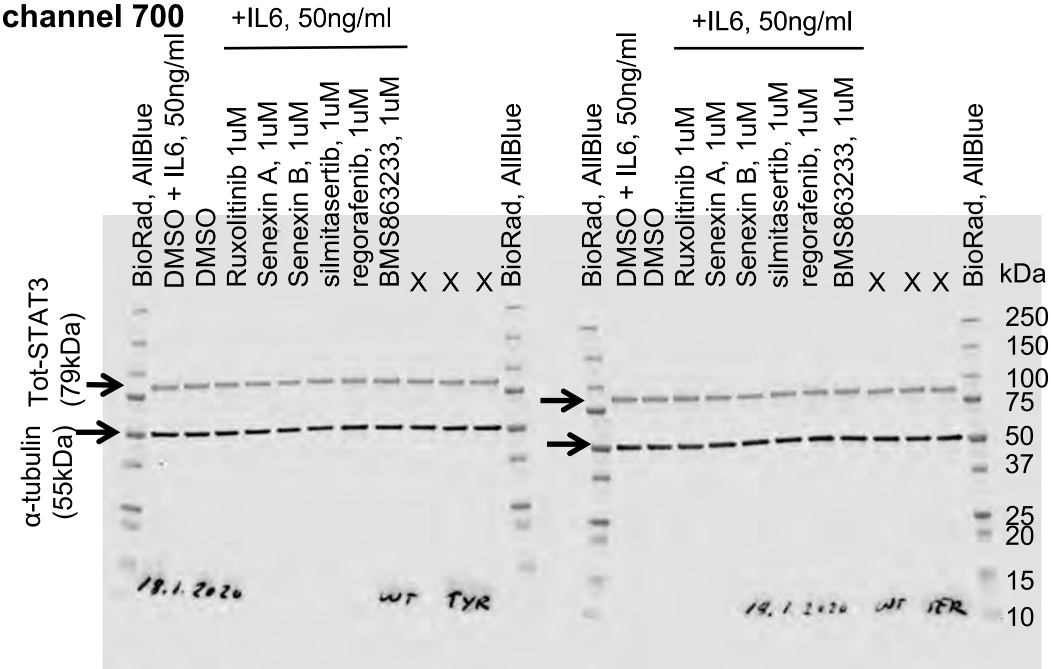

Overlay of two channels

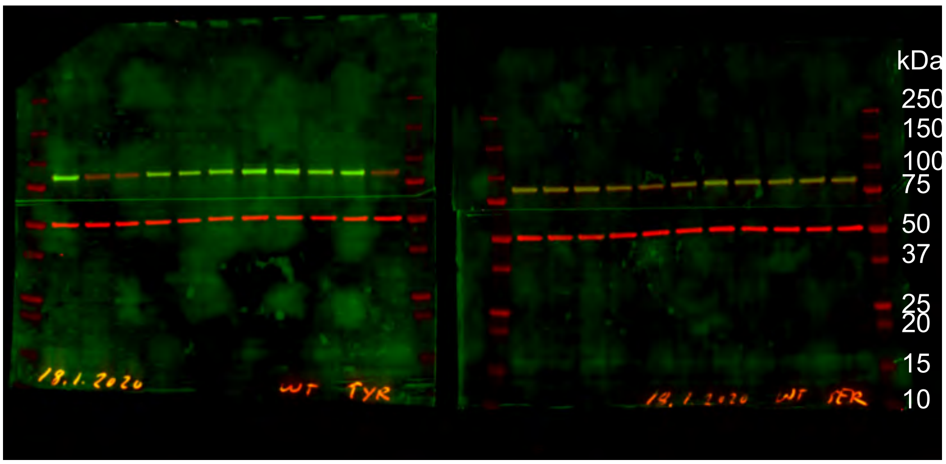

Raw images: Figure 5, analysis

Intensity 6, channel 800

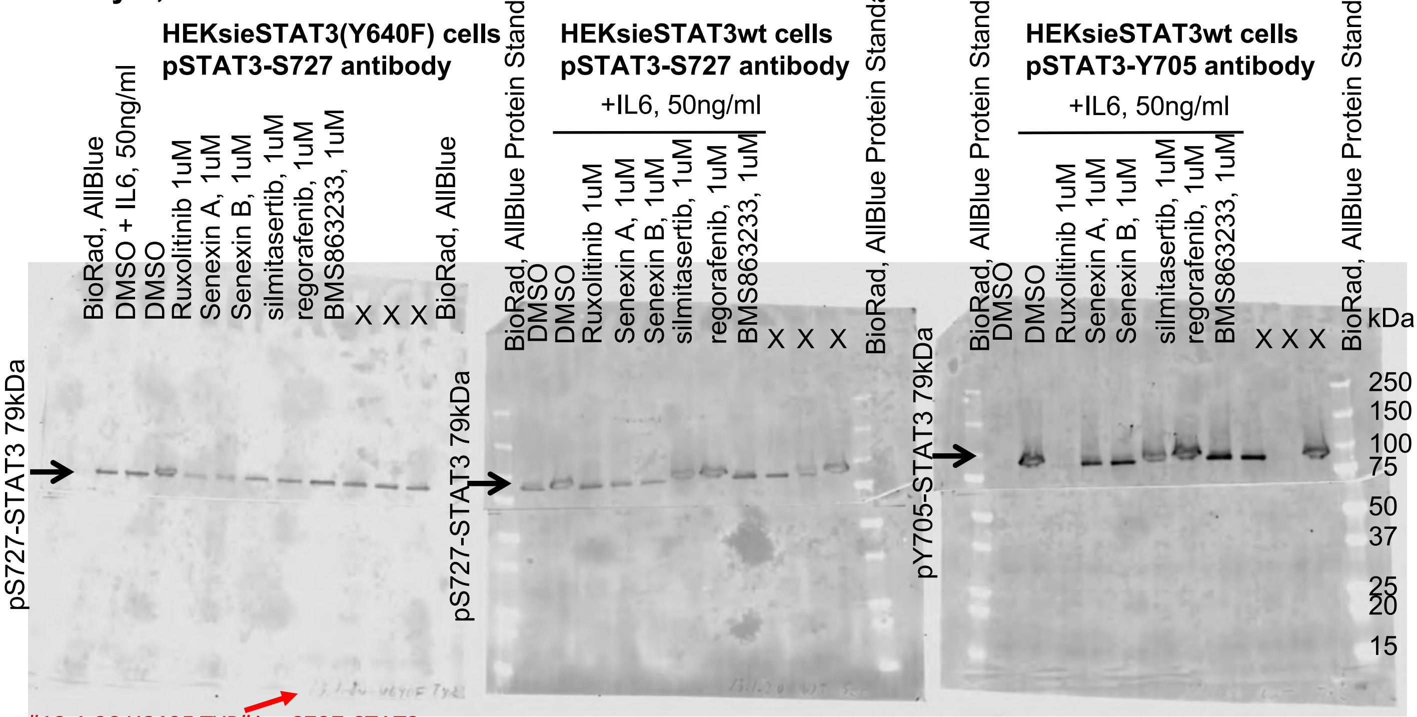

"13.1.20 Y640F TYR" is pS727-STAT3 stained membrane. Wrong hand written labelling on the actual membrane.

Intensity 5, channel 700

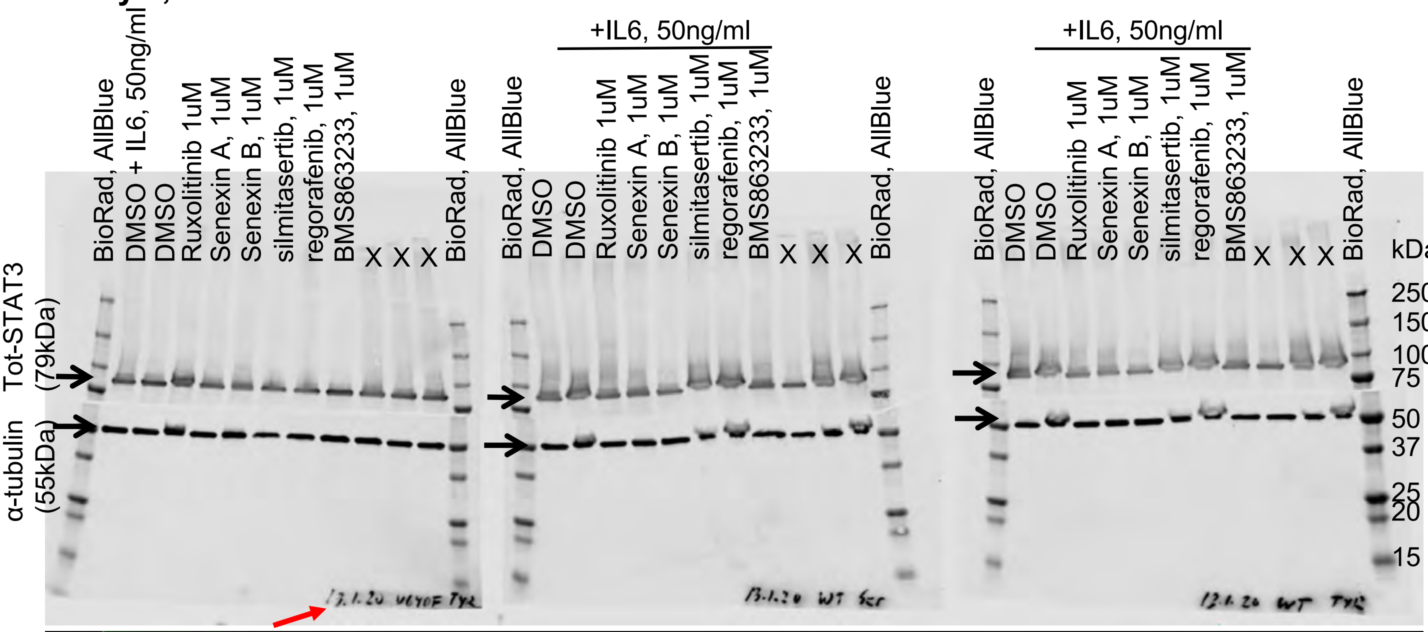

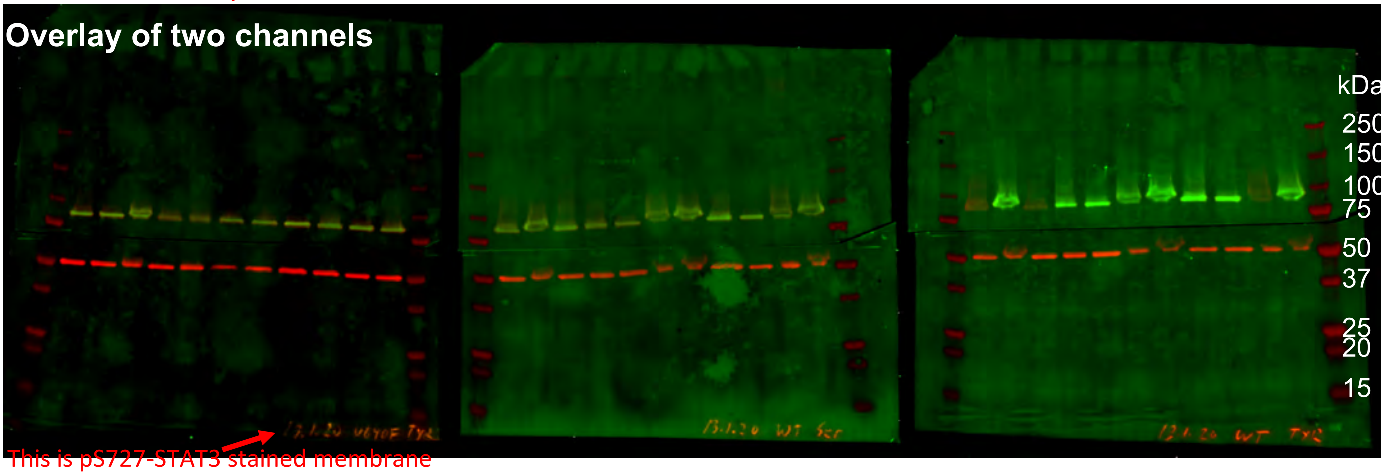

This is pS727-STAT3 stained membrane (green channel).
