# Supplementary material for: Identification of novel regulators of STAT3 activity
Source: PLoS One. 2020 Mar 31;15(3):e0230819. doi: 10.1371/journal.pone.0230819 (PMC7108870; doi:10.1371/journal.pone.0230819)
Supplement: Attachment — Submitted filename: PONE-D-19-33342 JD.docx [file pone.0230819.s010.docx]

In these experiments, the authors have searched for previously unexplored druggable targets that inhibit STAT3 signalling, in cells with both WT and mutant STAT3. They show that constitutively active STAT3 protects cells from cytotoxic responses to several

drug classes. They use transcription reporters in cells expressing STAT3 WT and STAT3(Y640F) in an RNAi screen, and identified kinases that altered activity. They use small molecule inhibition of several of kinase hits, which also blocked STAT3 activity, and conclude that targeting these kinases may therefore be an alternative strategy to inhibit STAT3 in cancers and other pathological conditions.

Comments:

The Introduction is informative with a nice rational, and its ends with a brief description of type of experiments. Previous work on drug sensitivity of STAT3 is referenced well. The experimental results are described in detail and look reasonable, but need a better take-away and what is the further work needed. Its is not clear what is novel in this study. In the discussion, they note that “in the siRNA screen three kinases and one phosphatase were identified (CDC7,415 PI4KII, DDR2 and PTPRH), which have not previously been linked directly to STAT3”. A number of other hits were discussed as previously identified regulators of STAT3, which adds confidence to their iRNA screen. However, drug screening does not appear to yield specificity for inhibition of activated STAT3(Y640F) compared to IL6 stimulated WT, nor for viability of STAT3(Y640F) cancer vs normal cells. The manuscript should be edited for clarity in this regard.

Other comments:

- The Discussion is long and the 1^st^ paragraph repeats previous ideas from the intro.
- There too much description of the result in the Figure legends, which are in the Results section. Figure Legends should be concise, noting the method, and any specifics; the number biological and technical repeats and statistical significance and method.
- How may independent experiments and what do error bars represent?
- The Figure legends should all be together after the references in manuscripts.
- Fig 2B “at least” is 2 words.
- Fig 2A and B could be simply described in the methods, as they are repetitive and contain a lot of text.
